# Structure-function analysis of MmpL7-mediated lipid transport in mycobacteria

**DOI:** 10.1016/j.tcsw.2021.100062

**Published:** 2021-08-31

**Authors:** Nabiela Moolla, Rebeca Bailo, Robert Marshall, Vassiliy N. Bavro, Apoorva Bhatt

**Affiliations:** a School of Biosciences and Institute of Microbiology and Infection, University of Birmingham, Edgbaston, Birmingham B15 2TT, UK; b School of Life Sciences, University of Essex, Colchester CO4 3SQ, UK

**Keywords:** *Mycobacterium tuberculosis*, MmpL, PDIM, Lipid transport, RND transporters

## Abstract

Mycobacterial membrane protein Large (MmpL7) is a Resistance-Nodulation-Division (RND) family transporter required for the export of the virulence lipid, phthiocerol dimycocerosate (PDIM), in *Mycobacterium tuberculosis*. Using a null mutant of the related, vaccine strain *Mycobacterium bovis* BCG, we show that MmpL7 is also involved in the transport of the structurally related phenolic glycolipid (PGL), which is also produced by the hypervirulent *M. tuberculosis* strain HN878, but absent in *M. tuberculosis* H37Rv. Furthermore, we generated an *in silico* model of *M. tuberculosis* MmpL7 that revealed MmpL7 as a functional outlier within the MmpL-family, missing a canonical proton-relay signature sequence, suggesting that it employs a yet-unidentified mechanism for energy coupling for transport. In addition, our analysis demonstrates that the periplasmic porter domain 2 insert (PD2-insert), which doesn't share any recognisable homology, is highly alpha-helical in nature, suggesting an organisation similar to that seen in the hopanoid PD3/4 domains. Using the *M. bovis* BCG *mmpL7* mutant for functional complementation with mutated alleles of *mmpL7*, we were able to identify residues present in the transmembrane domains TM4 and TM10, and the PD2 domain insert that play a crucial role in PDIM transport, and in certain cases, biosynthesis of PDIM.

## Introduction

The cell envelopes of mycobacteria, including the tuberculosis-causing *Mycobacterium tuberculosis*, are rich in unique lipids that form a hydrophobic barrier, playing a role in resistance and virulence (Jankute et al., 2015, Gago et al., 2018, Garcia-Vilanova et al., 2019).

**Fig. 1:**
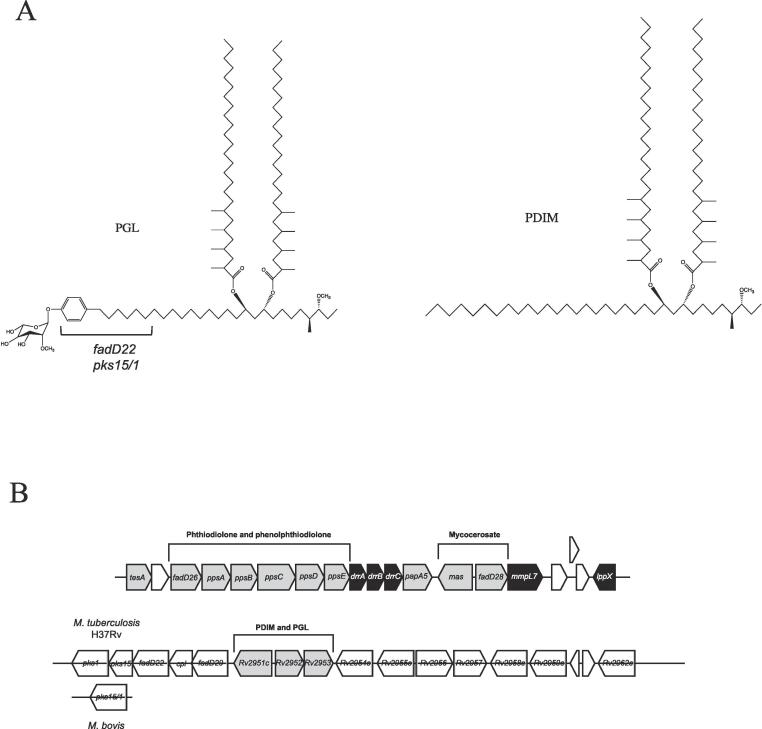
(A) Structures of Phthiocerol dimycocerosate (PDIM) and phenolic glycolipid (PGL). Enzymes responsible for the biosynthesis of the *p*-hydroxyphenylalkanoate moiety of PGLs are indicated (B) Map of the PDIM/PGL biosynthesis and transport cluster in *M. tuberculosis* H37Rv. *M. bovis* BCG contains a single ORF representing *pks1* and *pks15*.

Two *M. tuberculosis* cell envelope lipids, phthiocerol dimycocerosate (PDIM) and phenolic glycolipid (PGL) play an important role in virulence. PDIM and PGL share a common structural core, a diol backbone (phthiocerol), esterified with two methyl-branched fatty-acyl moieties (dimycocerosates) (Onwueme et al., 2005) (Fig. 1). Genes required for the biosynthesis of this core are present in a cluster that includes five Type-I polyketide synthases, PpsA-E, which synthesise phthiocerol. Additionally, a Type-I fatty acid synthase termed mycocerosic acid synthase (Mas) produces mycocerosic acid (Onwueme et al., 2005) that is eventually esterified to phthiocerol. The structure of PGL builds on this common lipidic core, further expanded by a phenolic group, and glycosylation (Constant et al., 2002, Jackson et al., 2021, Perez et al., 2004). The polyketide synthase Pks15/1 and a *p*-hydroxybenzoyl-AMP-ligase (FadD22) catalyse the formation of long-chain p-hydroxyphenylalkanoate intermediates that primes the synthesis of PGLs (Fig. 1). The *M. tuberculosis* strain H37Rv has a frameshift mutation in *pks15/1* resulting in a deficiency in PGL production (Constant et al., 2002, Reed et al., 2004)

PDIM has been shown to be required at different stages of infection and mutants deficient in PDIM biosynthesis or transport are attenuated in animal models of infection (Camacho et al., 2001, Cox et al., 1999, Rens et al., 2021). PGLs are associated with the hypervirulence of select *M. tuberculosis* strains such as those from the W-Beijing family (Constant et al., 2002, Reed et al., 2004). The critical role of these PDIMs and PGLs in virulence and immunomodulation, warrants a detailed study of how they are made, and subsequently exported to the outer envelope of mycobacteria.

Transport proteins termed Mycobacterial Membrane Protein Large (MmpL), are involved in the translocation of mycobacterial lipids across the plasma membrane (Chalut, 2016, Jackson et al., 2021). MmpLs are members of the Resistance-Nodulation-Division (RND) family of membrane proteins, that are thought to be powered by proton motive force (PMF) and are distinguished by a common architecture consisting of 12 transmembrane (TM) spanning regions and 2 porter domains (PD1 and PD2) formed out of large periplasmic loops located between TM1/2 and TM7/8 respectively (Jackson et al., 2021).

In *M. tuberculosis* the *mmpL7* (*Rv2942*) gene is located in the PDIM/PGL biosynthesis cluster (Onwueme et al., 2005) (Fig. 1). Null mutants of *M. tuberculosis mmpL7* accumulate PDIM intracellularly (Camacho et al., 2001, Cox et al., 1999). Furthermore, the PD2 of *M. tuberculosis* MmpL7 has been shown to interact with the phthiocerol-producing PpsE, suggesting that biosynthesis of PDIM may be coupled with its transport (Jain and Cox, 2005). Other lipid-transporting MmpLs appear to function as stand-alone transporters, with no other genes in the biosynthesis cluster linked to transport (Bailo et al., 2015, Chalut, 2016). In contrast, MmpL7 appears to operate within a wider scaffold of translocator proteins. Mutants of *M. tuberculosis lppX* (encoding a lipoprotein) and of the ABC-transporter *drrABC*, which is present in the same PDIM/PGL cluster, are also defective in PDIM-translocation, suggesting a complex and distinct transport mechanism, possibly driven by a multiple, interacting components (Camacho et al., 2001, Sulzenbacher et al., 2006). Also, uniquely, *M. tuberculosis* MmpL7 and its orthologues are the only MmpLs that do not possess conserved Asp/Tyr-residues located on the transmembrane helices 4/10 (TM4/TM10), critical for proton coupling of transport (Bernut et al., 2016, Chalut, 2016).

Furthermore, while MmpL7 is known to transport PDIMs, its role in the transport of PGLs, which share a common structural core, remains to be studied in the *M. tuberculosis* complex, though the transport of PGLs in *Mycobacterium marinum* has been inferred to be linked to MmpL7 (Cambier et al., 2014, Yu et al., 2012). *Mycobacterium bovis* BCG MmpL7 shares 100% amino acid identity with *M. tuberculosis* MmpL7, and has an intact *pks1/15* gene required for PGL-biosynthesis (Reed et al., 2004), allowing us to assess the role of MmpL7 in PGL-transport in slow growing mycobacteria.

Additionally, we aimed to study structure–function relationships of *M. tuberculosis* MmpL7 by a combination of *in silico* and experimental approaches. We used a *Mycobacterium bovis* BCG *mmpL7* null-mutant for complementation studies with mutant alleles of *M. tuberculosis mmpL7*, and to assess the impact of loss of *mmpL7* on PGL transport.

## Results, materials and discussion

**Fig. 2:**
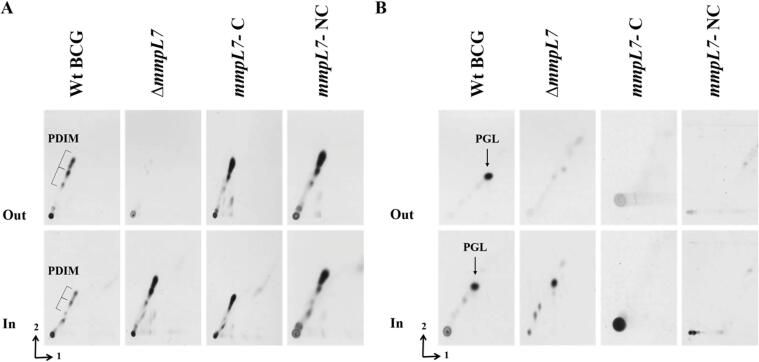
2D TLC autoradiography of [14C]-propionate-labelled lipids from Wild type (WT), Δ*mmpL7* mutant and two *mmpL7* complemented strains (Δ*mmpL7*-C contains pMV261-*mmpL7* and Δ*mmpL7*-NC containing pMV306-*mmpL7*). Apolar lipid extracts from the culture filtrate (Out) and intracellular lipids (In) were separated using (A) solvent systems A and (B) solvent system C (right). The three-pointed bracket shows the PDIM species, while the arrow depicts PGL. System A: direction 1, petroleum ether (60–80 °C): ethyl acetate, 95:2 (v/v) X 3; direction 2, Petroleum ether (60–80 °C): acetone 92:8 (v/v). System C: direction 1, chloroform: methanol 96:4 (v/v), direction 2, Toluene: acetone, 80:20 (v/v).

We generated a *M. bovis* BCG *mmpL7* null-mutant (Δ*mmpL7*) using Specialized Transduction (Bardarov et al., 2002, Larsen et al., 2007) and labelled cultures of wild type (WT) strain and Δ*mmpL7* mutant with ^14^C-propionate to selectively label methyl-branched fatty acid-containing lipids (including PDIMs and PGLs). Apolar lipids from spent media (exported lipids) and cell pellets (intracellular lipids) were extracted using standard methods (Dobson, 1985). Lipids were analysed by two-dimensional TLC systems A and C designed to visualise PDIMs and PGLs respectively (Dobson, 1985) (Fig. 2). As expected, the mutant showed loss of PDIM export and an intracellular accumulation of PDIMs (Fig. 2A) in agreement with the role of MmpL7 in *M. tuberculosis* (Camacho et al., 2001, Cox et al., 1999). A similar phenotype was observed for PGLs in the Δ*mmpL7*; PGL was not exported and accumulated intracellularly, implying MmpL7 was involved in the shared transport of PDIMs and PGLs (Fig. 2B). To complement the *M. bovis* BCG Δ*mmpL7* mutant, we cloned PCR-amplified *mmpL7* downstream of the constitutive *hsp60* promoter in the plasmid vector pMV261 (Stover et al., 1991) to generate the complementing plasmid pMV261-*mmpL7.* Transport of PDIMs was restored on transformation of Δ*mmpL7* with pMV261-*mmpL7* (Strain Δ*mmpL7-*C in Fig. 2A). Surprisingly, the complemented strain, which restored PDIM transport, showed a complete loss of PGLs. To account for the possibility that the overexpression of *mmpL7* in the complemented strain abrogated PGL synthesis, *mmpL7* was additionally cloned into the integrative vector pMV306 (Stover et al., 1991) under its native promoter to allow for generation of a separate complemented strain with a single copy and native expression patterns of *mmpL7*. This new strain, referred to as *mmpL7*-NC, showed a phenotype similar to that of Δ*mmpL7*-C*,* with PDIM-transport being restored, but PGL-biosynthesis being abolished (Fig. 2A, B).

To probe this further, we sequenced the two complemented strains. No mutations were observed in PDIM-biosynthesis genes, however both complemented strains harboured a mutation in *fadD22* which, as mentioned above, encodes a *p*-hydroxybenzoyl-AMP-ligase that primes PGL biosynthesis (Simeone et al., 2010). Accumulation of PGLs intracellularly could be toxic in the long term (prolonged subculture and selection) and the *fadD22* mutation likely represents a compensatory mutation.

**Fig. 3:**
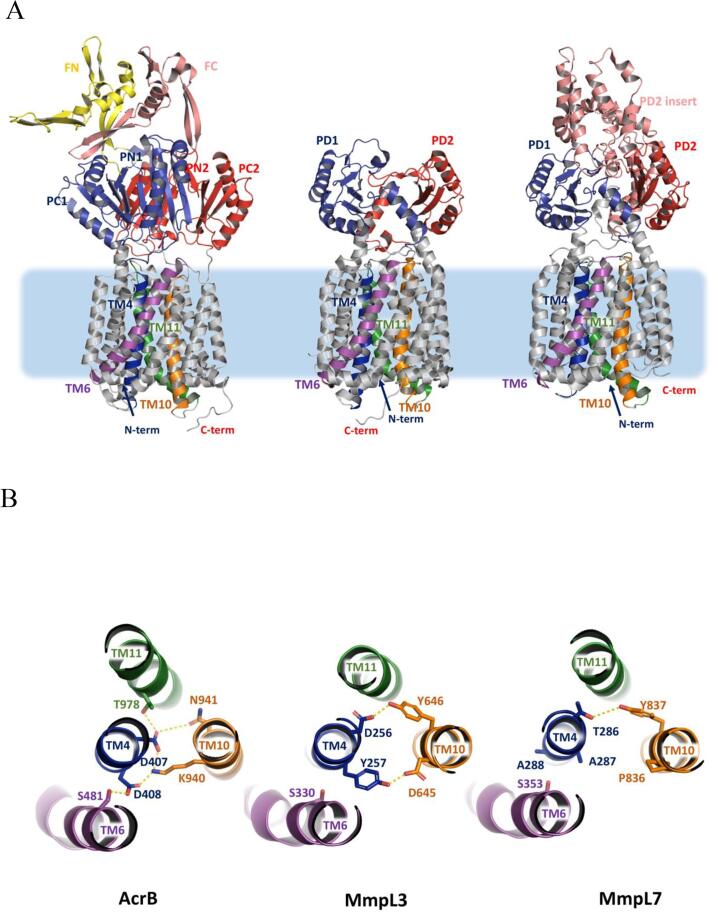
(A) Comparison of the structural organisation of a typical RND-transporter (with the example of the *E. coli* AcrB; 2GIF.pdb), the MmpL3 transporter (experimental structure 6OR2.pdb, missing the C-terminal D3 domain), and MmpL7 (homology model, this study), highlighting the commonality of transmembrane (TM) domain assembly. Transmembrane helices TM4, TM6, TM10 and TM11 are coloured in deep blue, magenta, orange and green respectively. (B) Top-down view of a section of the TM-domain of the corresponding structures centred around the residues forming the proton-relays (AcrB, MmpL3), and the equivalent area in MmpL7, displaying lack of conservation and residues unsuitable to proton transfer. (For interpretation of the references to colour in this figure legend, the reader is referred to the web version of this article.)

To identify and study functional domains, we generated a homology model of MmpL7 using the I-TASSER server (Yang et al., 2015), using structurally characterised RND-transporters, the *E.coli* AcrB (Johnson et al., 2020, Nakashima et al., 2011, Seeger et al., 2006) and *M. smegmatis* MmpL3 (Su et al., 2019, Zhang et al., 2019), as templates (Fig. 3A). As the latter is the closest relative of MmpL7 of known structure (See File S1, Supplementary Information for a multiple sequence alignment), with 14.7% identity, and 27% sequence similarity, we used it for primary homology modelling. It has to be noted that due to the low overall sequence similarity the predictive power of any such homology models is limited, so in addition, to avoid model bias and to compare the packing of the conserved TM-regions, we also used the AcrB as a reference RND-structure.

**Fig. 4:**
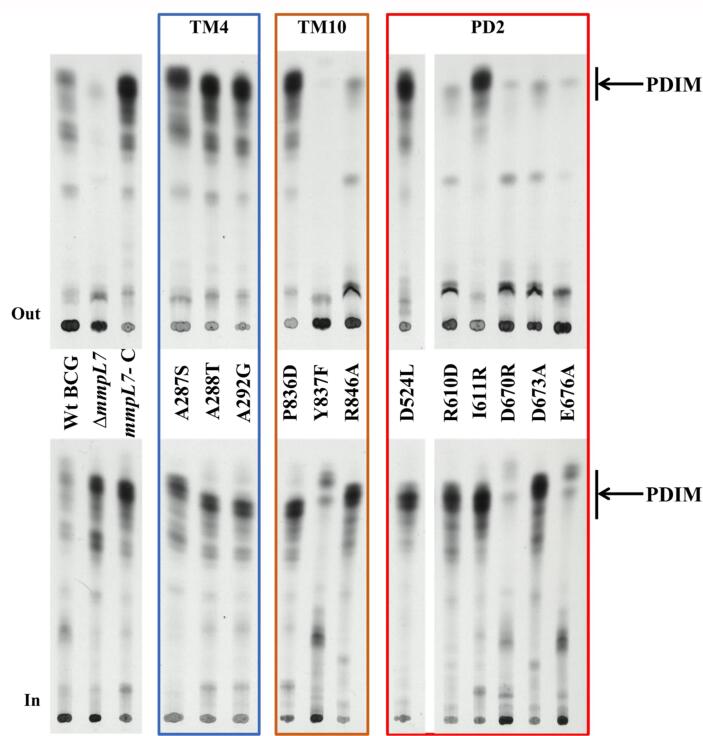
PDIM analysis of *mmpL7* SDM mutants. TLC autoradiography of [^14^C]-propionate labelled lipids from Wild type (Wt), Δ*mmpL7* mutant and thirteen *mmpL7* complemented strains. Amino acid substitutions (and position) are indicated between the two TLC plates. Apolar lipid extracts from the culture filtrate (Out) and intracellular lipids (In) were separated using petroleum ether: diethyl ether (9:1 v/v). An arrow shows the PDIM species.

In RND-permeases the energy required for substrate antiport is derived from proton-gradients stored across the inner membrane, which are channelled *via* conserved residues situated in TM4 and TM10 (Alav et al., 2021, Goldberg et al., 1999, Guan and Nakae, 2001), forming a proton-relay (Fig. 3B), which in MmpLs are represented by Asp/Tyr-pairs (Bernut et al., 2016, Park et al., 2015, Zhang et al., 2019). While the homology model of MmpL7 suggested that it shares most of its structural features with the other MmpLs (File S1, Supplementary Material), superimpositions at conserved proton-relay sites revealed differences. Namely, two Ala-residues (Ala287/288) are present on TM4, while Pro836/Tyr837 are found in place of the Asp-Tyr pair on TM10 conserved in all other MmpLs (Fig. 3; Files S1 and S2, Supplementary Material) (Bernut et al., 2016, Chalut, 2016, Melly and Purdy, 2019). Furthermore, closer inspection of the sequence of MmpL7 did not reveal any Asp-Tyr or indeed any residue-pairs capable of forming a proton relay within its TM-domain. In the same TM10-helix, we identified another residue (Arg846) conserved across MmpL7-proteins from obligate pathogens (File S2, Supplementary Material), not mentioned in previous works. Further analysis suggests that the majority of the residues forming the RND proton-relay network have no direct correspondence in MmpL7. On the other hand, analysis of MmpL7 orthologues from PDIM-producing mycobacteria reveals conservation of TM4 Ala287 and TM10 Pro836 (Fig. 3B). We targeted these residues to probe whether they contributed, in a yet unknown mechanism, to MmpL7 activity (File S2, Supplementary Material). To that end, the replicative pMV261 complementing plasmid-derivative containing *mmpL7*, was used as a template for site-directed mutagenesis and mutated alleles were then transformed into the BCG Δ*mmpL7*-strain to test the ability to restore PDIM-transport. *mmpL7* expression was confirmed in all transformed strains by RT-PCR (File S3, Supplementary Material). Most of the mutated *mmpL7-*alleles were able to complement the null-mutant, restoring PDIM-transport, suggesting that these conserved residues were not critical for function (Fig. 4). Two separate mutations did affect the ability to restore PDIM-transport: the R846A substitution affected the ability to translocate PDIMs, while Y837F completely abolished PDIM-biosynthesis (Fig. 4). The loss of PDIM-biosynthesis in the latter is significant as whole genome sequencing did not reveal any mutations in PDIM-biosynthesis genes, indicating that the observed loss is due to the altered MmpL7. Thus, Y837 appears to play a wider-role, perhaps in coordinating PDIM-biosynthesis with transport.

**Fig. 5:**
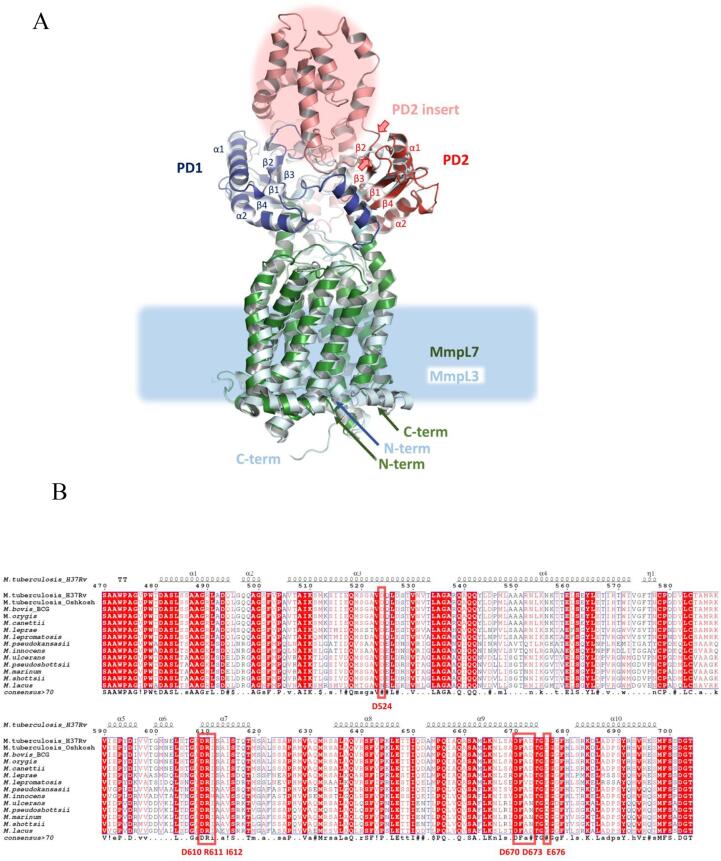
(A) Side view of the superposition of the 3D structures of the MmpL3 (cyan, based on 6OR2.pdb) and MmpL7 (homology model). Key domains in MmpL7 are coloured green (TM), blue (PD1) and red (PD2) respectively, with the PD2-insertion (indicated by the two red arrows) in salmon. Both PD1 and PD2 domains have a common architecture, with secondary structure elements being numbered. The analysis of the model reveals high general similarity of TM and periplasmic architecture between MmpL3 and MmpL7, with the exception of the large PD2-domain insertion, which is not present in MmpL3. (B) Multiple sequence alignment of the MmpL7 orthologues, visualised using Espript3, highlighting the predicted highly alpha-helical nature of the PD2-insertion and conservation of its residues across mycobacteria. The residues mutated in this study have been highlighted with red boxes. (For interpretation of the references to colour in this figure legend, the reader is referred to the web version of this article.)

RND-transporters have a common structural blueprint shared across the family members, including MmpLs and are composed of several conserved modules (Alav et al., 2021). The TM4/TM10, which couple the proton-motive force with substrate efflux, transmit conformational changes to the periplasmic porter domains, which provide substrate-binding and dictate specificity (Su et al., 2019) and, in Gram-negative bacteria interact with adaptor proteins (Symmons et al., 2015) (Fig. 3A). These porter domains are themselves formed on a modular principle, based on a core module presenting a mixed α-β sandwich of a general (β1 − α1 − β2 − β3 − α2 − β4) configuration dubbed the PD-module (Alav et al., 2021). Each periplasmic loop of the MmpLs contains a single PD-module, known as PD1 and PD2, for the N-terminal and C-terminal lobe of the transporter respectively (Chim et al., 2015, Zhang et al., 2019) (Fig. 5A). Consistent with that, comparative analysis between the MmpL7 homology-model and MmpL3 PD-domains revealed near identical PD1 shared between them, but a distinct PD2, which in the case of MmpL7 contains an additional structural motif, spliced between β2 − β3 strands (Fig. 5A). Further comparison with the AcrB PD-domains, reveals that while the PD1-domain of MmpL7 is similar to the contiguous AcrB PN1/PC1-domains, the PD2-domain presents an insertion, at an equivalent position (namely between the respective β2 − β3 strands) to the insertion of the FN (funnel or docking) sub-domains within the PN2/PC2 seen in the AcrB (Murakami et al., 2002). While the insertions are taking place at equivalent, conserved positions, which in the MmpL7 correspond to residues A472-T704 inclusive (Alav et al., 2021), our analysis indicates that there is no similarity between the MmpL7 PD2-inserts and AcrB FN-domains (Fig. 3; Fig. 5A). Indeed, as indicated by a BLASTp search against the PDB-database, the MmpL7 PD2 insert does not share recognisable homology with any known structures, but is predicted to be strongly alpha-helical in nature, possibly forming helical bundles, similar to the organisation of the hopanoid PD3/4 domains, which are inserted into the PD1 and PD2 of HpnN in an equivalent fashion (Alav et al., 2021, Kumar et al., 2017).

In MmpL7 the PD2-domain has been identified as critical for recruitment of PpsE, the last polyketide synthase to produce phthiodiolone in PDIM in *M. tuberculosis* (Fig. 1B) (Jain and Cox, 2005), suggesting that the PD2-domains in MmpLs may be involved in protein–protein interactions. There are few lines of evidence that support this hypothesis. The lipid-binding lipoprotein LpqN, has been reported to interact with the related MmpL3 and MmpL11 (Melly et al., 2019), facilitating the passage of exported lipid to the outer envelope. Furthermore, LpqN-family proteins LpqT and Mtc28, as well as the non-homologous lipoprotein LprG (Rv1411c), interact with the MmpL11 PD2-domain, but not the MmpL3 (Melly et al., 2019), with the latter being able to bind phosphatidylinositol-containing glycolipids and interact with the mycolyl transferase, Ag85A (Drage et al., 2010, Melly et al., 2019). Intriguingly, LprG and LppX belong to the same family of homologous lipid-binding lipoproteins, that in addition includes two lipoproteins of yet-unknown function, LprA and LprF. It is thus possible that LppX-MmpL7 interaction is similar to that observed between the LprG-MmpL11.

These findings from previous work strongly suggest that the PD2-domains of MmpL proteins, and specifically their insertions, are likely focal points for scaffolding association of enzymatic machineries involved in the lipid-modification. Due to the lack of reliable structural template for the PD2-inserts, rational mutagenesis is problematic, but we probed several residues within MmpL7-PD2, conserved across MmpL7-orthologues (namely Asp524, Arg610, Ile611 and Asp670) (Fig. 4B) for functional effects. Additionally, we tested Asp673 and Glu676, which are found in the MmpL7-orthologues of obligatory pathogens and could likely have a bespoke role in this group of mycobacteria (Fig. 4B).

All substitutions apart from R610D and D673A reinstated PDIM-transport (Fig. 4). However, the *M. bovis* BCG Δ*mmpL7*-strains carrying the D670R or E676A-substitutions showed complete loss of PDIM-biosynthesis, similar to what we observed in some of the above TM10-substitutions (e.g. Y837F). Notably both Ile611 and Asp673 were previously shown to be essential for the MmpL7-PpsE interaction (Jain and Cox, 2005). Similarly, whole-genome sequencing of the transformed strains did not reveal any mutations in PDIM-biosynthesis genes, suggesting a wider, yet-unidentified role for these residues. Consistent with the possible role of these residues in protein–protein interactions, within our I-TASSER model, the R610D and D637A map to the top of the helical PD2-insertion domain (File S4, panel A, Supplementary Material).

While this work was under review, we gained access to the AlphaFold v2.0 predictions (Jumper et al., 2021) for MmpL7 (https://alphafold.ebi.ac.uk/entry/P9WJU7; File S4, Supplementary Material). In contrast to traditional homology modelling, AlphaFold v.2.0 uses a contextual *ab initio* prediction minimising model bias. We anticipated this to be of particular benefit for the interpretation of the mutations within the PD2-insert of MmpL, which is hindered by the lack of reliable homology templates and based on the available structural data, our only reliable predictions were of high alpha-helical propensity of the insert. The PD2-insert of the AlphaFold model is consistent with our prediction, being fully alpha-helical and forming a helical bundle, but it has to be noted that it has also the lowest confidence prediction score within overall model, which limits its predictive power. While the overall conformation of the PD2-insert is markedly different, the locations of the phenotypically pronounced mutations are consistent with the PD2-domain playing a docking-platform for protein–protein interactions (File S4, panel A, Supplementary Material). Furthermore, encouragingly, the results from AlphaFold v2.0, show close convergence with our earlier models within the TM and PD1/PD2 section of the protein with an RMSD of < 5 Å over Cα-backbone, excluding the PD2-insert. There are subtle, yet significant differences in the packing of TM10 and 11, with TM 11 packing tightly between TM4 and TM10, and segregating the conserved Tyr837 from its canonical partnering residues in TM4, further confirming that a canonical proton relay with the participation of the Tyr837 is not possible, which is consistent with our mutagenesis results (File S4, panel B, Supplementary Material). Intriguingly, this alternative conformation of the Tyr837 brings it in the vicinity of a cluster of charged residues located at the cytoplasmic end of TM11 (Arg863) and TM (Gln909; Arg910), however future work would be required to elucidate the exact mechanism of energy coupling of MmpL7.

In summary, our findings identify MmpL7 as a functional outlier within the MmpL-family, with a distinctive amino-acid signature with its TM-domain, suggesting a yet-unidentified mechanism for energy coupling. Furthermore, we have identified the alpha-helical PD2-insert domain of MmpL7 to be critical for its function. Given the role of other proteins in PDIM transport, it is likely that MmpL7 is part of a much larger complex for PDIM and PGL transport, likely providing a scaffold for co-ordinating transport with biosynthesis these virulence lipids. While the *in silico* model of MmpL7 used in this study is not without limitations, it helps to inform future structural and functional studies of this medically important protein.

## Declaration of Competing Interest

The authors declare that they have no known competing financial interests or personal relationships that could have appeared to influence the work reported in this paper.

## References

[b0005] Alav I., Kobylka J., Kuth M.S., Pos K.M., Picard M., Blair J.M.A., Bavro V.N. (2021). Structure, assembly, and function of tripartite efflux and type 1 secretion systems in gram-negative bacteria. Chem. Rev..

[b0010] Bailo R., Bhatt A., Ainsa J.A. (2015). Lipid transport in Mycobacterium tuberculosis and its implications in virulence and drug development. Biochem. Pharmacol..

[b0015] Bardarov S., Bardarov S., Pavelka M.S., Sambandamurthy V., Larsen M., Tufariello J., Chan J., Hatfull G., Jacobs W.R. (2002). Specialized transduction: an efficient method for generating marked and unmarked targeted gene disruptions in Mycobacterium tuberculosis, M. bovis BCG and M. smegmatis. Microbiology.

[b0020] Bernut A., Viljoen A., Dupont C., Sapriel G., Blaise M., Bouchier C., Brosch R., de Chastellier C., Herrmann J.L., Kremer L. (2016). Insights into the smooth-to-rough transitioning in Mycobacterium bolletii unravels a functional Tyr residue conserved in all mycobacterial MmpL family members. Mol. Microbiol..

[b0025] Camacho L.R., Constant P., Raynaud C., Laneelle M.A., Triccas J.A., Gicquel B., Daffe M., Guilhot C. (2001). Analysis of the phthiocerol dimycocerosate locus of Mycobacterium tuberculosis. Evidence that this lipid is involved in the cell wall permeability barrier. J. Biol. Chem..

[b0030] Cambier C.J., Takaki K.K., Larson R.P., Hernandez R.E., Tobin D.M., Urdahl K.B., Cosma C.L., Ramakrishnan L. (2014). Mycobacteria manipulate macrophage recruitment through coordinated use of membrane lipids. Nature.

[b0035] Chalut N. (2016). MmpL transporter-mediated export of cell-wall associated lipids and siderophores in mycobacteria. Tuberculosis (Edinb).

[b0040] Chim N., Torres R., Liu Y., Capri J., Batot G., Whitelegge J.P., Goulding C.W. (2015). The structure and interactions of periplasmic domains of crucial mmpl membrane proteins from mycobacterium tuberculosis. Chem. Biol..

[b0045] Constant P., Perez E., Malaga W., Laneelle M.A., Saurel O., Daffe M., Guilhot C. (2002). Role of the pks15/1 gene in the biosynthesis of phenolglycolipids in the Mycobacterium tuberculosis complex. Evidence that all strains synthesize glycosylated p-hydroxybenzoic methyl esters and that strains devoid of phenolglycolipids harbor a frameshift mutation in the pks15/1 gene. J. Biol. Chem..

[b0050] Cox, J. S., CHEN, B., MCNEIL, M. & JACOBS, W. R., JR. 1999. Complex lipid determines tissue-specific replication of Mycobacterium tuberculosis in mice. Nature, 402, 79-83. 10.1038/47042 10573420

[b0055] Dobson G, Minnikin D.E., Minnikin S.M., Parlett M., Goodfellow M. & M., R. 1985. Systematic analysis of complex mycobacterial lipids. Chemical Methods in Bacterial Systematics. . London: Academic Press.

[b0060] Drage M.G., Tsai H.C., Pecora N.D., Cheng T.Y., Arida A.R., Shukla S., Rojas R.E., Seshadri C., Moody D.B., Boom W.H., Sacchettini J.C., Harding C.V. (2010). Mycobacterium tuberculosis lipoprotein LprG (Rv1411c) binds triacylated glycolipid agonists of Toll-like receptor 2. Nat. Struct. Mol. Biol..

[b0065] Gago G., Diacovich L., Gramajo H. (2018). Lipid metabolism and its implication in mycobacteria-host interaction. Curr Opin Microbiol.

[b0070] Garcia-Vilanova A., Chan J., Torrelles J.B. (2019). Underestimated manipulative roles of mycobacterium tuberculosis cell envelope glycolipids during infection. Front. Immunol..

[b0075] Goldberg M., Pribyl T., Juhnke S., Nies D.H. (1999). Energetics and topology of CzcA, a cation/proton antiporter of the resistance-nodulation-cell division protein family. J. Biol. Chem..

[b0080] Guan L., Nakae T. (2001). Identification of essential charged residues in transmembrane segments of the multidrug transporter MexB of Pseudomonas aeruginosa. J. Bacteriol..

[b0085] Jackson M., Stevens C.M., Zhang L., Zgurskaya H.I., Niederweis M. (2021). Transporters Involved in the biogenesis and functionalization of the mycobacterial cell envelope. Chem. Rev..

[b0090] Jain M., Cox J.S. (2005). Interaction between polyketide synthase and transporter suggests coupled synthesis and export of virulence lipid in M. tuberculosis. PLoS Pathog..

[b0095] Jankute M., Cox J.A., Harrison J., Besra G.S. (2015). Assembly of the mycobacterial cell wall. Annu. Rev. Microbiol..

[b0100] Johnson R.M., Fais C., Parmar M., Cheruvara H., Marshall R.L., Hesketh S.J., Feasey M.C., Ruggerone P., Vargiu A.V., Postis V.L.G., Muench S.P., Bavro V.N. (2020). Cryo-EM structure and molecular dynamics analysis of the fluoroquinolone resistant mutant of the AcrB transporter from salmonella. Microorganisms.

[b0105] Jumper John, Evans Richard, Pritzel Alexander, Green Tim, Figurnov Michael, Ronneberger Olaf, Tunyasuvunakool Kathryn, Bates Russ, Žídek Augustin, Potapenko Anna, Bridgland Alex, Meyer Clemens, Kohl Simon A.A., Ballard Andrew J., Cowie Andrew, Romera-Paredes Bernardino, Nikolov Stanislav, Jain Rishub, Adler Jonas, Back Trevor, Petersen Stig, Reiman David, Clancy Ellen, Zielinski Michal, Steinegger Martin, Pacholska Michalina, Berghammer Tamas, Bodenstein Sebastian, Silver David, Vinyals Oriol, Senior Andrew W., Kavukcuoglu Koray, Kohli Pushmeet, Hassabis Demis (2021). Highly accurate protein structure prediction with AlphaFold. Nature.

[b0110] Kumar N., Su C.C., Chou T.H., Radhakrishnan A., Delmar J.A., Rajashankar K.R., Yu E.W. (2017). Crystal structures of the Burkholderia multivorans hopanoid transporter HpnN. Proc Natl Acad Sci U S A.

[b0115] Larsen, M.H., Biermann, K., Tandberg, S., Hsu, T. & Jacobs, W.R., JR. 2007. Genetic Manipulation of Mycobacterium tuberculosis. Curr Protoc Microbiol, Chapter 10, Unit 10A 2. 10.1002/9780471729259.mc10a02s6 18770603

[b0120] Melly G., Purdy G.E. (2019). MmpL proteins in physiology and pathogenesis of M. tuberculosis. Microorganisms.

[b0125] Melly G.C., Stokas H., Dunaj J.L., Hsu F.F., Rajavel M., Su C.C., Yu E.W., Purdy G.E. (2019). Structural and functional evidence that lipoprotein LpqN supports cell envelope biogenesis in Mycobacterium tuberculosis. J. Biol. Chem..

[b0130] Murakami S., Nakashima R., Yamashita E., Yamaguchi A. (2002). Crystal structure of bacterial multidrug efflux transporter AcrB. Nature.

[b0135] Nakashima R., Sakurai K., Yamasaki S., Nishino K., Yamaguchi A. (2011). Structures of the multidrug exporter AcrB reveal a proximal multisite drug-binding pocket. Nature.

[b0140] Onwueme K.C., Vos C.J., Zurita J., Ferreras J.A., Quadri L.E. (2005). The dimycocerosate ester polyketide virulence factors of mycobacteria. Prog. Lipid Res..

[b0145] Park I.K., Hsu A.P., Tettelin H., Shallom S.J., Drake S.K., Ding L., Wu U.I., Adamo N., Prevots D.R., Olivier K.N., Holland S.M., Sampaio E.P., Zelazny A.M. (2015). Clonal diversification and changes in Lipid Traits and Colony Morphology in Mycobacterium abscessus clinical isolates. J. Clin. Microbiol..

[b0150] Perez E., Constant P., Lemassu A., Laval F., Daffe M., Guilhot C. (2004). Characterization of three glycosyltransferases involved in the biosynthesis of the phenolic glycolipid antigens from the Mycobacterium tuberculosis complex. J. Biol. Chem..

[b0155] Reed M.B., Domenech P., Manca C., Su H., Barczak A.K., Kreiswirth B.N., Kaplan G., Barry C.E., 3RD (2004). A glycolipid of hypervirulent tuberculosis strains that inhibits the innate immune response. Nature.

[b0160] Rens C., Chao J.D., Sexton D.L., Tocheva E.I., Av-Gay Y. (2021). Roles for phthiocerol dimycocerosate lipids in Mycobacterium tuberculosis pathogenesis. Microbiology (Reading).

[b0165] Seeger M.A., Schiefner A., Eicher T., Verrey F., Diederichs K., Pos K.M. (2006). Structural asymmetry of AcrB trimer suggests a peristaltic pump mechanism. Science.

[b0170] Simeone R., Leger M., Constant P., Malaga W., Marrakchi H., Daffe M., Guilhot C., Chalut C. (2010). Delineation of the roles of FadD22, FadD26 and FadD29 in the biosynthesis of phthiocerol dimycocerosates and related compounds in Mycobacterium tuberculosis. FEBS J..

[b0175] Stover C.K., de la Cruz V.F., Fuerst T.R., Burlein J.E., Benson L.A., Bennett L.T., Bansal G.P., Young J.F., Lee M.H., Hatfull G.F., & (1991). New use of BCG for recombinant vaccines. Nature.

[b0180] Su C.C., Klenotic P.A., Bolla J.R., Purdy G.E., Robinson C.V., Yu E.W. (2019). MmpL3 is a lipid transporter that binds trehalose monomycolate and phosphatidylethanolamine. Proc. Natl. Acad. Sci. U.S.A..

[b0185] Sulzenbacher G., Canaan S., Bordat Y., Neyrolles O., Stadthagen G., Roig-Zamboni V., Rauzier J., Maurin D., Laval F., Daffe M., Cambillau C., Gicquel B., Bourne Y., Jackson M. (2006). LppX is a lipoprotein required for the translocation of phthiocerol dimycocerosates to the surface of Mycobacterium tuberculosis. EMBO J..

[b0190] Symmons M.F., Marshall R.L., Bavro V.N. (2015). Architecture and roles of periplasmic adaptor proteins in tripartite e ffl ux assemblies. Front. Microbiol..

[b0195] Yang J., Yan R., Roy A., Xu D., Poisson J., Zhang Y. (2015). The I-TASSER suite: protein structure and function prediction. Nat. Methods.

[b0200] Yu J., Tran V., Li M., Huang X., Niu C., Wang D., Zhu J., Wang J., Gao Q., Liu J. (2012). Both phthiocerol dimycocerosates and phenolic glycolipids are required for virulence of Mycobacterium marinum. Infect. Immun..

[b0205] Zhang B., Li J., Yang X., Wu L., Zhang J., Yang Y., Zhao Y., Zhang L., Yang X., Yang X., Cheng X., Liu Z., Jiang B., Jiang H., Guddat L.W., Yang H., Rao Z. (2019). Crystal structures of membrane transporter MmpL3, an anti-TB drug target. Cell.

