# Supplementary material for: Structure-function analysis of MmpL7-mediated lipid transport in mycobacteria
Source: Cell Surf. 2021 Aug 31;7:100062. doi: 10.1016/j.tcsw.2021.100062 (PMC8427324; doi:10.1016/j.tcsw.2021.100062)
Supplement: Supplementary data 1 [file mmc1.pdf]

Fig S1

A multiple sequence alignment of the MmpL family members (paralogues) within *M. tuberculosis* visualised using Espript3 highlighting their common organisation. The top sequence and secondary structure is derived from the experimentally determined *M. smegmatis* MmpL3 structure. Principal secondary structure elements are annotated above the sequences, including transmembrane helices (TM) 1-12; the PD1 and PD2 periplasmic domains, as well as the skirting helices and C-terminal helix. The long C-terminal extensions seen in MmpL3 (beyond residue 750) constitute an additional D3 domain. Red boxes highlight conserved residues in TM4 and TM10 implicated in proton-relay function. TM helices participating in the formation and support of the proton relay are colour coded as explained in Fig 2.

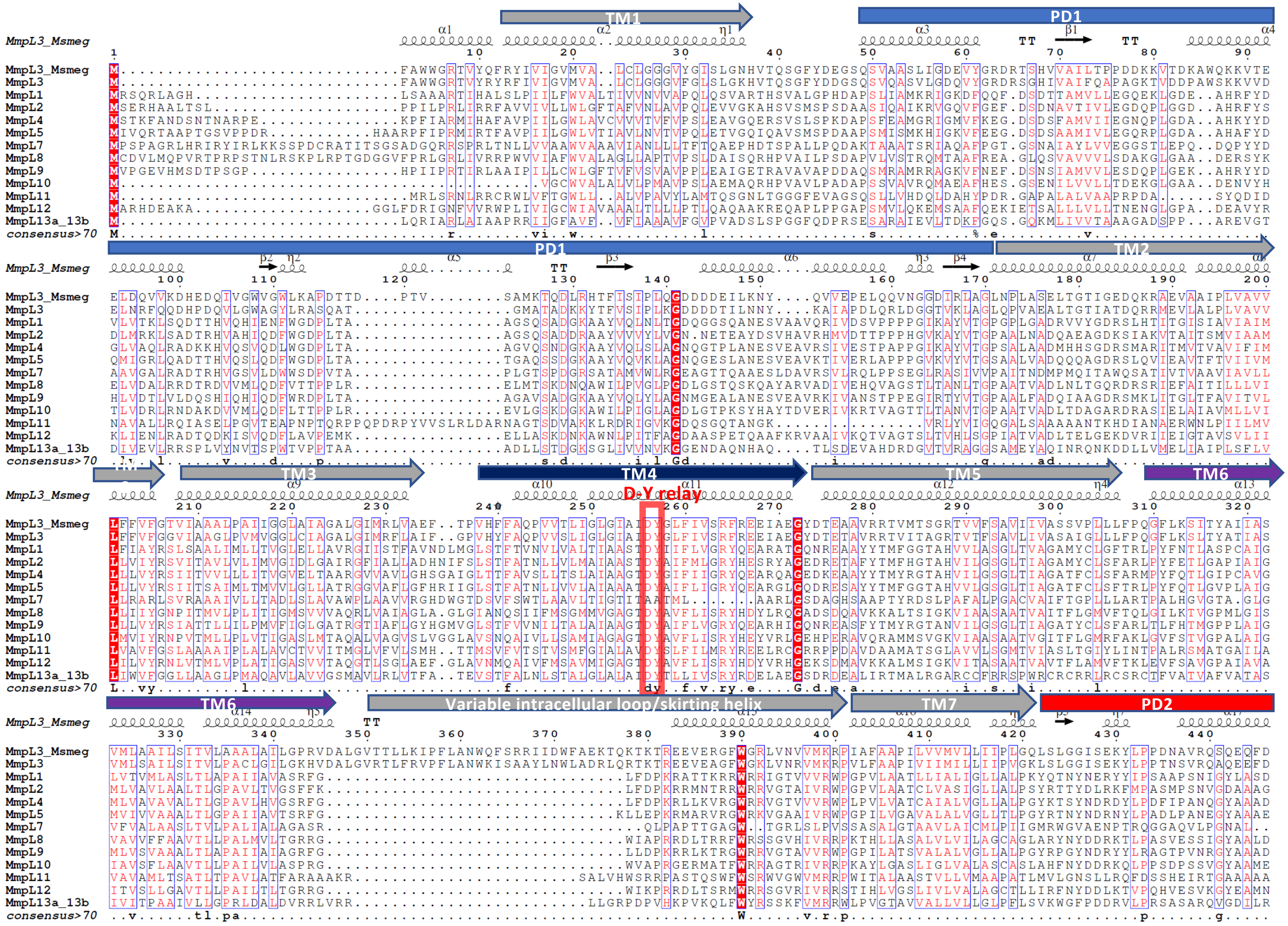

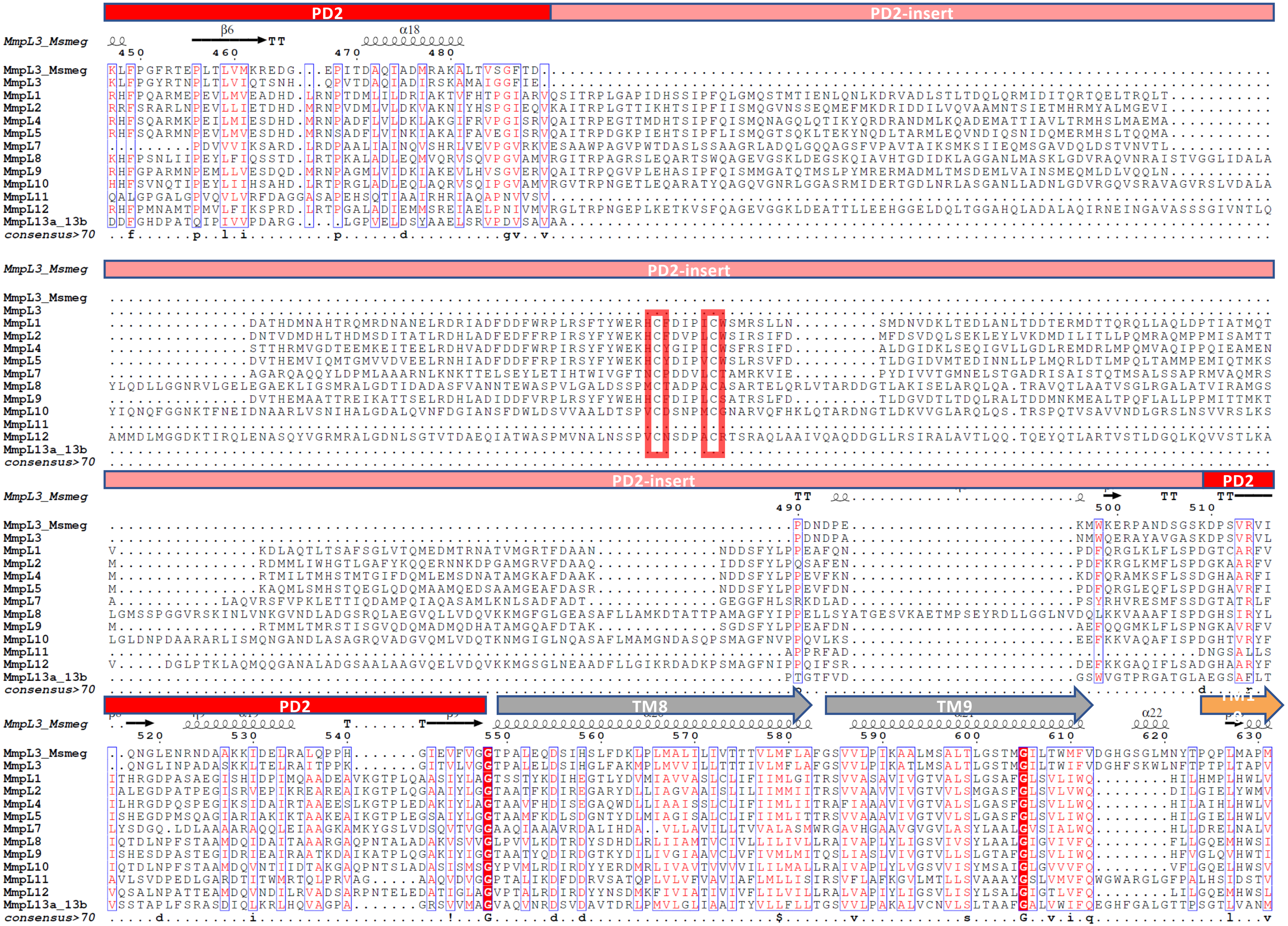
PD2
PD2-insert
TM8
TM9
TM1
β6
α18
α22
MmpL3_Msmeg
MmpL3
MmpL1
MmpL2
MmpL4
MmpL5
MmpL7
MmpL8
MmpL9
MmpL10
MmpL11
MmpL12
MmpL13a_13b
consensus>70

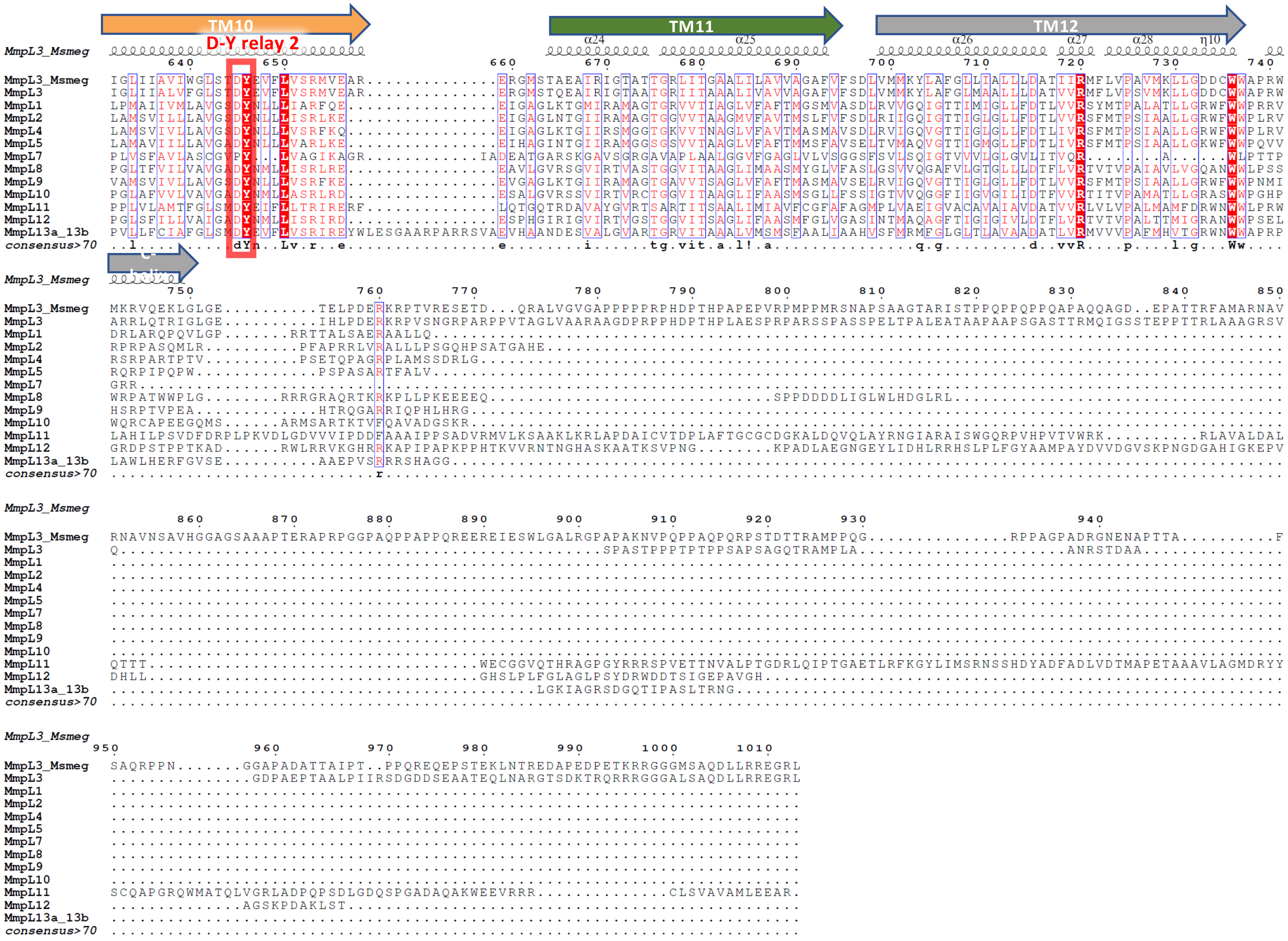

TM10
D-Y relay 2
TM11
TM12
C-helix
MmpL3_Msmeg
MmpL3
MmpL1
MmpL2
MmpL4
MmpL5
MmpL7
MmpL8
MmpL9
MmpL10
MmpL11
MmpL12
MmpL13a_13b
consensus>70
