# Supplementary material for: Structure-function analysis of MmpL7-mediated lipid transport in mycobacteria
Source: Cell Surf. 2021 Aug 31;7:100062. doi: 10.1016/j.tcsw.2021.100062 (PMC8427324; doi:10.1016/j.tcsw.2021.100062)
Supplement: Supplementary data 2 [file mmc2.pdf]

File S2

A multiple sequence alignment of the MmpL7 orthologues across mycobacteria visualised using Espript3. The top sequence and secondary structure is derived from the homology model of MmpL7 discussed within the manuscript. Principal secondary structure elements are annotated above the sequences, including transmembrane helices (TM) 1-12; the PD1 and PD2 periplasmic domains, as well as the helical PD2-insertion (salmon). Red boxes highlight residues in TM4 and TM10 whose position corresponds to the conserved proton-relay forming residues in the other members of the MmpL family. The position of the R846, discussed in the text is also highlighted.

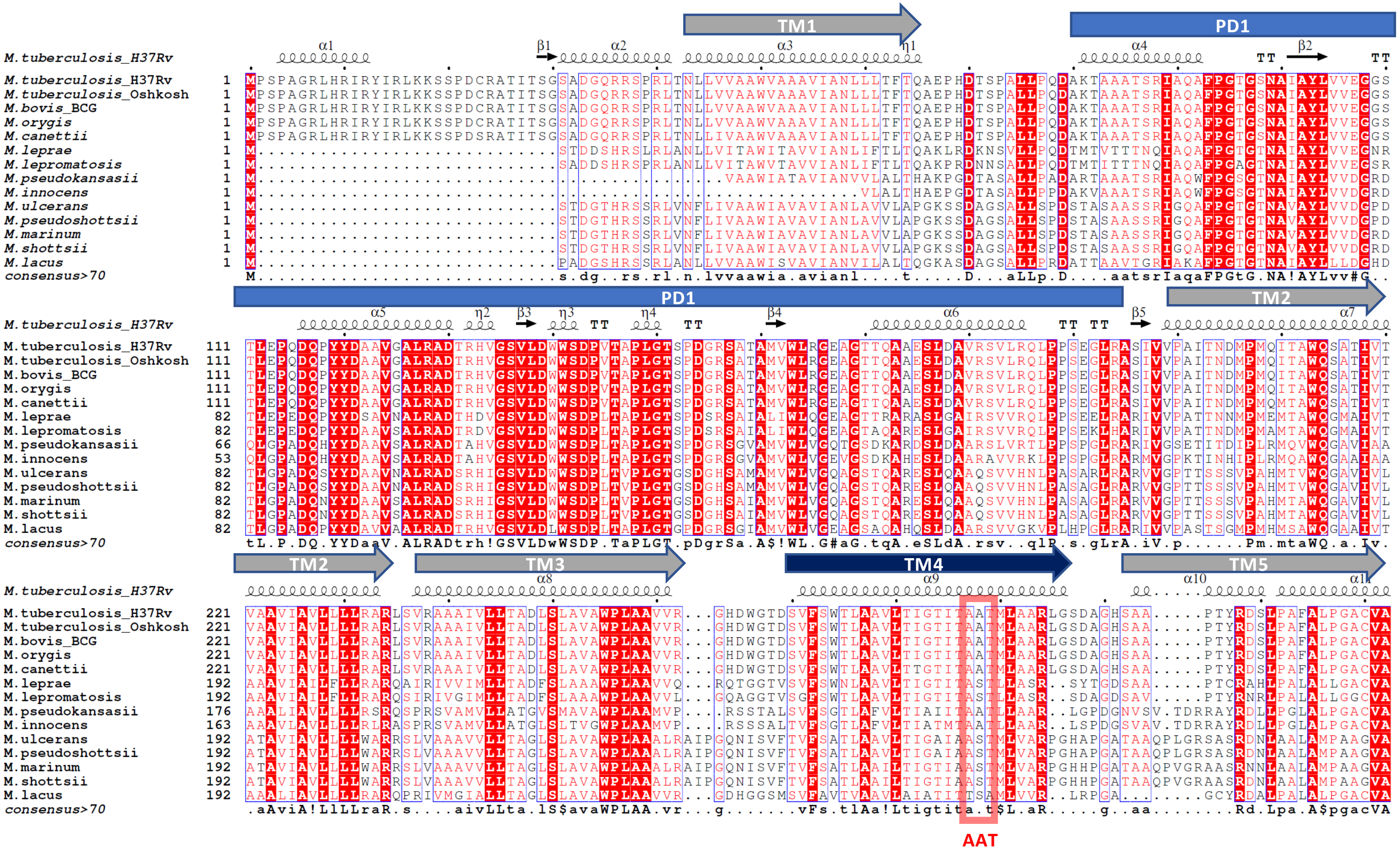

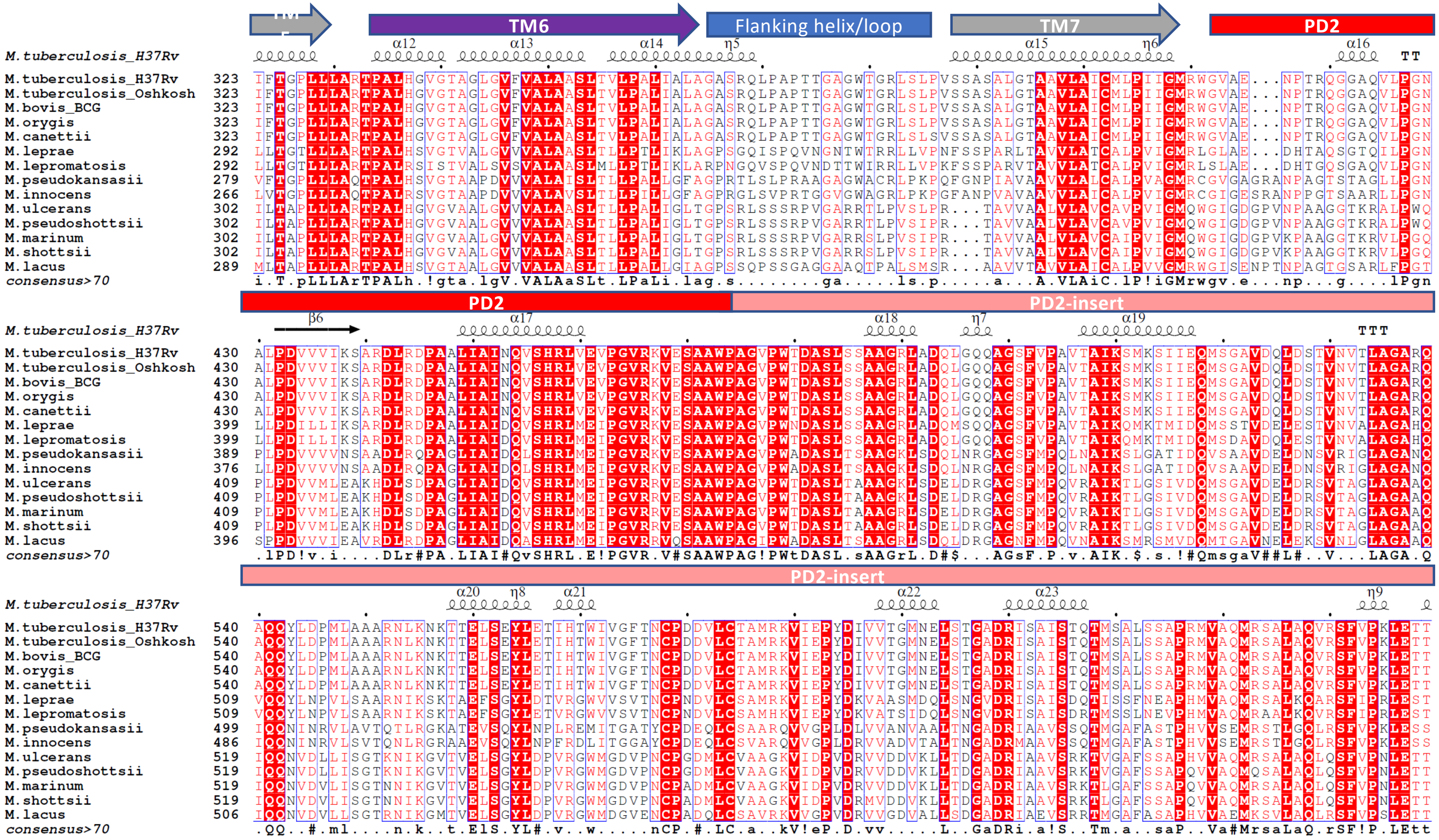
TM6
Flanking helix/loop
TM7
PD2
PD2-insert
M.tuberculosis_H37Rv
M.tuberculosis_Oshkosh
M.bovis_BCG
M.orygis
M.canettii
M.leprae
M.lepromatosis
M.pseudokansasii
M.innocens
M.ulcerans
M.pseudoshottsii
M.marinum
M.shottsii
M.lacus
consensus>70

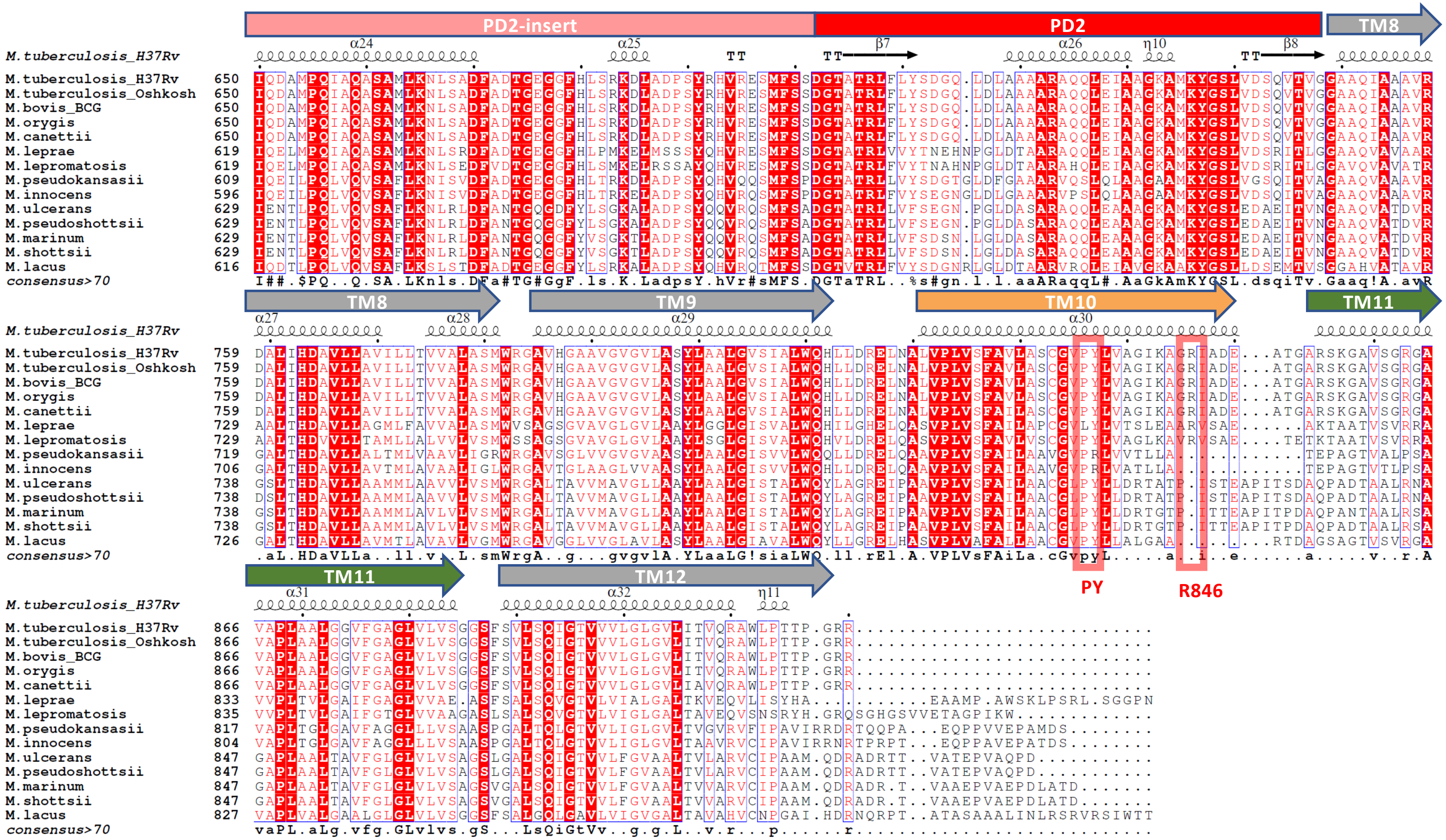
PD2-insert
PD2
TM8
TM9
TM10
TM11
TM12
PY
R846
M.tuberculosis_H37Rv
M.tuberculosis_Oshkosh
M.bovis_BCG
M.orygis
M.canettii
M.leprae
M.lepromatosis
M.pseudokansasii
M.innocens
M.ulcerans
M.pseudoshottsii
M.marinum
M.shottsii
M.lacus
consensus>70
