# Supplementary material for: Structure-function analysis of MmpL7-mediated lipid transport in mycobacteria
Source: Cell Surf. 2021 Aug 31;7:100062. doi: 10.1016/j.tcsw.2021.100062 (PMC8427324; doi:10.1016/j.tcsw.2021.100062)
Supplement: Supplementary data 3 [file mmc3.pdf]

File S3

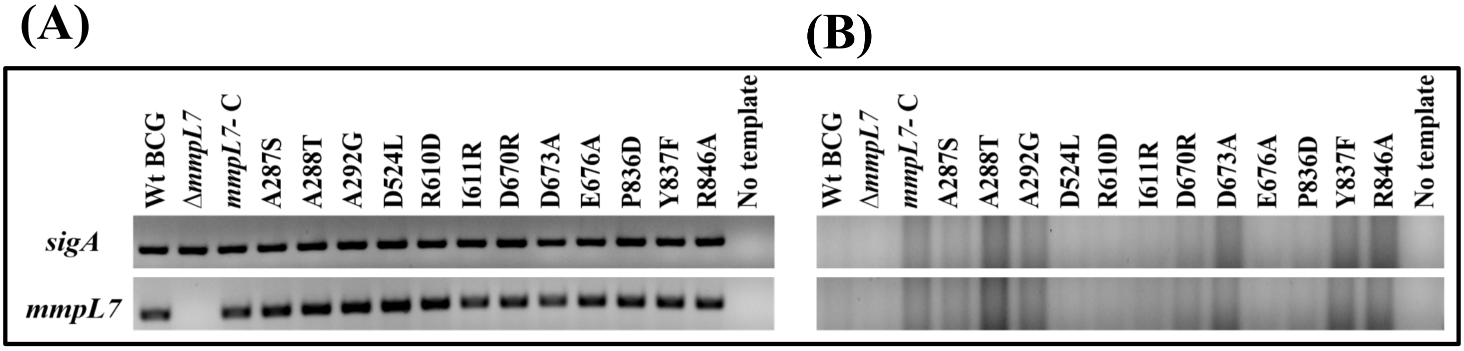

Reverse transcriptase- PCR analysis to confirm *mmpL7* expression. (A) Expression of the *mmpL7* gene in the wild type (Wt) BCG, the Δ*mmpL7* mutant and the thirteen *mmpL7* complemented strains as compared to the expression of the housekeeping gene *sigA* as a positive control. (B) Control reactions with no reverse transcriptase added.
