# Supplementary material for: Structure-function analysis of MmpL7-mediated lipid transport in mycobacteria
Source: Cell Surf. 2021 Aug 31;7:100062. doi: 10.1016/j.tcsw.2021.100062 (PMC8427324; doi:10.1016/j.tcsw.2021.100062)

File S4

(A) Comparison of the MmpL7 models generated by the *ab initio* prediction algorithm implemented in AlphaFold v.2.0 (right), vs the homology model generated by the I-TASSER discussed in this manuscript (left). (B) A comparison of the helical packing and residue interactions within the vicinity of the residues which in the other MmpL transporters have been implicated in formation of proton-relays involved in energy coupling of transport. The view corresponds to a top view (90° rotation), relative to panel A at the level of the dashed line.

A

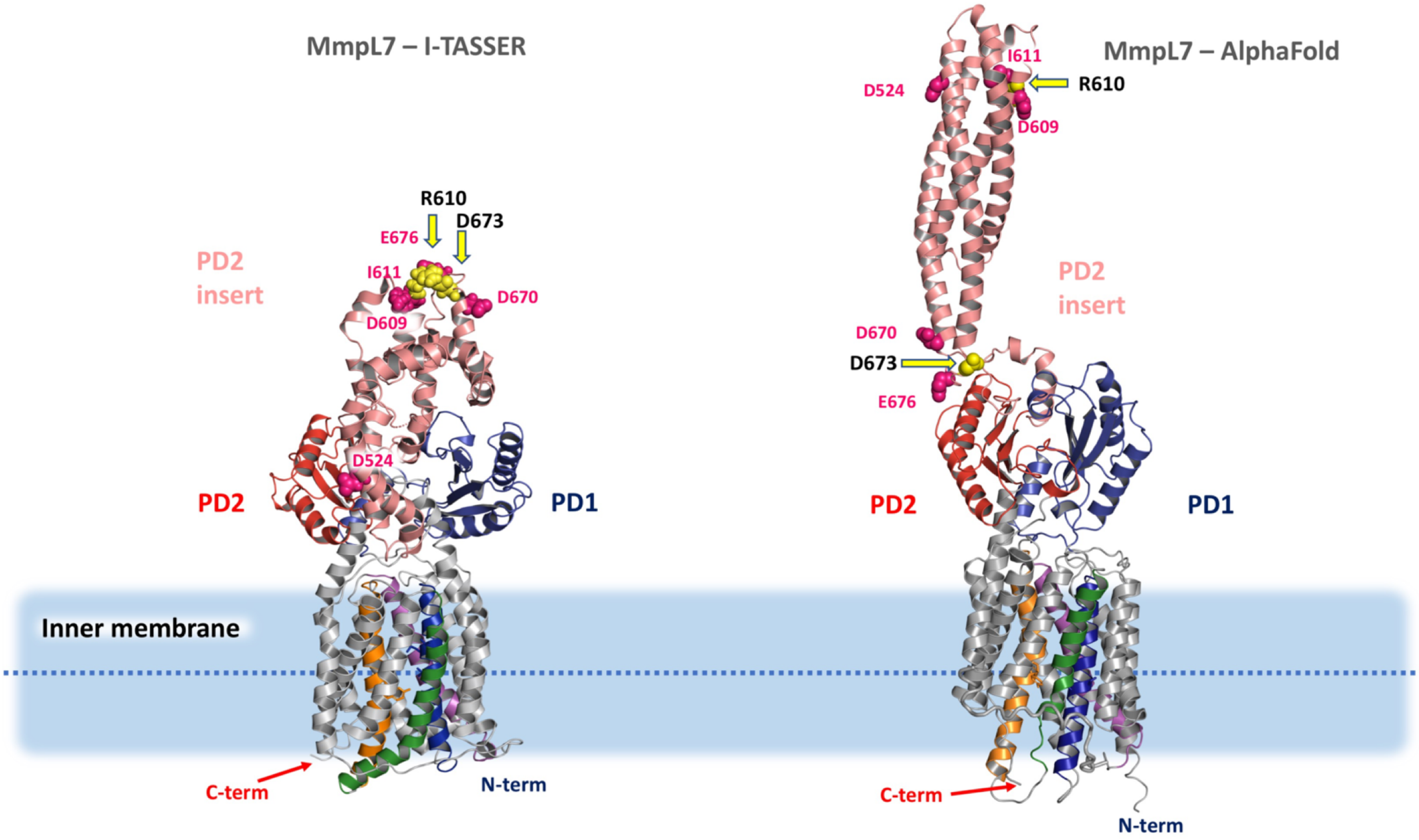

B

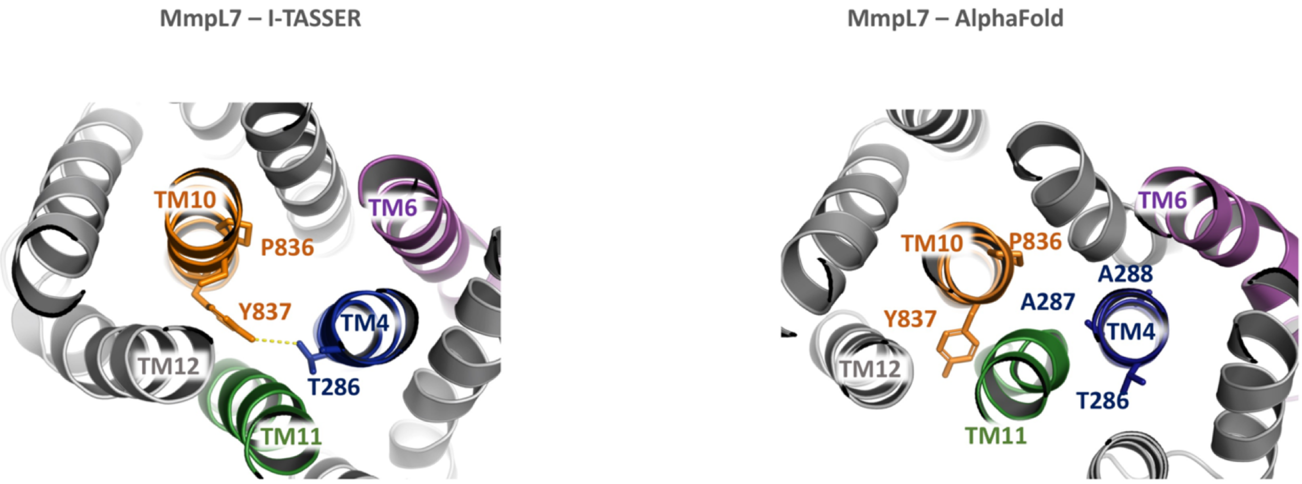

Supplement: Supplementary data 4 [file mmc4.pdf]
